# “I wanted to hide but also to be found”: the high school experiences of young adults who grew up in the same home as a sibling with depression

**DOI:** 10.1186/s40359-023-01234-y

**Published:** 2023-06-29

**Authors:** Inbar Levkovich, Michal Labes

**Affiliations:** Faculty of Graduate Studies, Oranim Academic College of Education, Kiryat Tiv’on, 36006 Israel

**Keywords:** Depression, Siblings, School, Depressive symptoms, Mental health, Qualitative research

## Abstract

**Background:**

Depression is a mental health condition that can have far-reaching consequences for the entire family, not just for the affected individual. Siblings are particularly vulnerable in that the unremitting stress and guilt at home can affect multiple aspects of their lives, including relationships, added responsibilities, and health. This pressure may affect siblings’ own emotional well-being and academic success. Most studies in this field have examined the impact of depression on the affected adolescents or their parents, whereas few have examined the impact on siblings. Sibling studies have been limited by lack of sample homogeneity, especially in the context of coping in high school. This study sought to examine the retrospective experiences of young adults who lived in the same house as a sibling with depression while they were in high school.

**Methods:**

This qualitative study examined 21 young adults (aged 18–29) who grew up with a sibling with depression. In-depth, semi-structured interviews were conducted from May to September 2022. The interviews were recorded and transcribed and underwent thematic analysis.

**Results:**

Three main themes emerged from the interviews: (1) “School as a place of refuge”: The perspective of participants who grew up with a sibling with depression regarding their high school experience. (2) “I wanted the adults at school to see me”: Relations between research participants and the school educational staff. (3) “I was afraid people would relate to me as the sibling of a crazy person”: Participants’ relationships with their peers.

**Conclusions:**

This study sheds light on the experiences of adolescents who grew up with a sibling with depression. The findings point to feelings of being invisible, self-nullification, avoiding sharing with others, and transparency. The participants were afraid that if their peers found out about their sibling they would also be stigmatized and alienated. The study shows that adolescents living with a sibling with depression need support at school.

**Supplementary Information:**

The online version contains supplementary material available at 10.1186/s40359-023-01234-y.

## Introduction

Depression is a prevalent psychiatric disorder that finds expression in depressed mood, loss of pleasure and interest, feelings of emptiness, sleep disruptions and loss of appetite [1]. Depression has consequences for the emotional, social, academic, and developmental circumstances of affected individuals and for their future [2, 3]. Moreover, it has far-reaching implications for the entire family, not just for the affected individual. Adolescents living in the same family with an individual with depression are at increased risk of becoming distressed and developing mental illnesses [4–6]. In general, empirical knowledge is lacking about the siblings of children with depression, and particularly about their high school experiences.

Sibling relationships are characterized by unique and significant dimensions that encompass shared life experiences, a common genetic background, and enduring connections throughout the life cycle [5]. These relationships involve varying degrees of intimacy and investment and provide opportunities for connection, validation, belonging, and mirrored experience, as well as for competition and distress [7]. When one sibling experiences mental illness, the effects on the remaining siblings can be profound and disruptive [8]. According to previous research, the siblings of children with mental health issues experience negative psychological effects, including, though not limited to, grief, anxiety, loneliness, jealousy, anger, and guilt [9, 10]. The adolescent siblings of someone with depression are prone to behavioral and emotional changes [11–14]. A study that examined adaptation among the adolescent siblings of someone diagnosed with mental illness found that 19% showed signs of depression and anxiety, 16% exhibited aggressive behavior, and 12% exhibited signs of rebellion and failure to conform to rules [4]. Review studies suggest that the siblings of children with mental health are at risk of developing mental health problems and are more likely to experience internalizing problems such as depression and anxiety than externalizing problems such as conduct issues [15–17].

Researchers have proposed several pathways to explain how one child’s mental illness may affect the mental health of his/her siblings [18]. The most prominent conceptual model in this field was developed by Wallander and Varni [19], who identified risk and protective factors that contribute to the psychosocial adjustment of siblings. This model comprises seven interconnected factor categories, each with numerous individual factors, including disease and disability (e.g., diagnosis, severity), functional independence (e.g., ambulation, communication), psychosocial stress (e.g., daily hassles), adjustment/adaptation (e.g., social, physical), personal factors (e.g., temperament, problem-solving skills), socio-ecological factors (e.g., family members’ adjustment, social support), and stress processing (e.g., coping strategies, cognitive appraisal). For instance, previous research has indicated that the psychosocial adjustment of the siblings of children with chronic health conditions is closely related to the impact of the child’s illness on day-to-day functioning [20]. This relationship is attributed to increased caretaking responsibilities, related psychosocial stress, and decreased parental attention and family relationship problems that are often reported by siblings [21].

These findings are reinforced by other studies showing that the adolescent siblings of a child with mental illness are liable to exhibit more behavioral problems and difficulties in regulating their emotions than a control group [4, 6]. They may also be forced to assume additional caregiving and domestic responsibilities and may witness repeated acute medical situations, which can result in differential treatment within the family [22]. because they feel they must help their brother or sister cope with depression, such that their school achievements are liable to be affected [23–25]. Many studies indicate that individuals living with a sibling with mental illness do their utmost to compensate for their sibling’s shortcomings [11, 12], for example by assuming the burden of care, often at the price of self-sacrifice and self-nullification [11, 26].

Moreover, a sibling’s depression is liable to have an impact on adolescents’ relationships with their peer group and their teachers. The family members of someone with a mental illness tend to avoid telling others about their family situation [14]. The reasons for this concealment are varied: fear of violating the sibling’s privacy by revealing personal information, fear of negative stigmas being assigned to them or their families, or fear of being labeled as socially deviant [14, 27]. A study that examined the experiences of adolescents living with a sibling diagnosed with a mental illness revealed feelings of being rejected by their surroundings, a loss of social status, feelings of lack of self-worth, and negative stigmas assigned to their family [27]. Concealing their sibling’s situation caused adolescents to feel isolated and cut off from sources of social support [28]. In contrast, adolescents living with a sibling with a mental disorder are also liable to develop the ability to be highly compassionate and empathetic [6].

The literature shows that psycho-educational intervention at school with the families of individuals with mental illness has a positive impact on adolescents’ high school experience. Studies showed that intervention reduced the number of depressive symptoms and improved academic achievements. Moreover, the intervention helped diminish difficult emotions such as shame and guilt, reinforced ties within the family, and prevented family conflicts from accelerating [29, 30].

### Theoretical framework

The Bowen family systems theory [31] is based on a dynamic perspective of the world, according to which everything that happens in the inner life of an individual within the family has a direct impact on the family atmosphere and the family members. Bowen claimed that individuals have less autonomy in their emotional lives than what they believe because they are dependent upon and respond to those around them. Hence, an individual within the family cannot be observed without observing the broader context [32].

Two basic concepts in Bowen’s theory are differentiation and fusion. Differentiation refers to the desire to be a separate individual with a clear and defined identity and sense of self who is different from the rest of the family to the point of separation. Fusion refers to the desire for close relations with the rest of the family, to the point of being swallowed up by the desires and needs of others [31]. The individual within the family is constantly activated by these two opposing forces. A relationship based on fusion is generally characteristic of families during the first years after the birth of a baby. In the normal process of maturation, these family ties become less close. Yet during crises a close relationship becomes essential [32]. Family ties that remain too close during adolescence are marked by dependence, intensity, and confusion regarding the roles of the family members [31, 32].

Nevertheless, very few studies have examined family system relationships in families in which one of the children is affected by depression [21, 32, 33]. Research indicates that the members of such families tend to express anger towards one another more often and that hostile sibling relationships and communication barriers with parents can exacerbate depressive symptoms [21, 33]. Furthermore, studies in the United States show that parental preference for one sibling has a negative impact on the sense of family support during crises, with support dwindling as parental preference for the depressed sibling increases. In contrast, warm sibling relationships that entail intimacy, support, and affection can diminish emotional distress for all siblings [32]. The current research, which is based on Bowen’s theory, offers a unique perspective by exploring the family dynamics of the adolescent siblings of children with depression. While most studies have focused on the experience of the depressed child or the parents, this research recognizes the significant impact that depression can have on siblings as well [34, 35]. By assuming that the experience of the depressed child and the parents will also affect the siblings living in the same home [21], this study sheds light on the complex interplay between family members and the ways in which mental health issues can affect the entire family unit [33].

The primary research question examined in this study is as follows:

- What are the experiences of young adults who lived with a sibling with depression while they were in high school?

The study also examined a number of secondary questions:

- How do the research participants remember their high school experiences as adolescents in the context of their sibling’s depression?
- How do the research participants remember their relations with the school educational staff in the context of their sibling’s depression?
- How do the research participants remember their relations with their peers in the context of their sibling’s depression?

## Methods

### Approach

To answer the research question, this study used the qualitative phenomenological method, thus attempting to gain a deep understanding of the phenomenon by immersing in the participants’ world and experiences [36]. This approach was chosen because it is well-suited for investigating the sibling experience, making it possible to explore the participants’ feelings, thoughts, insights, and understandings [37]. By utilizing this approach, the participants were able to share their stories and attribute meaning to their experiences, thereby facilitating a more comprehensive understanding of the phenomenon [36–38].

### Participants and sample

The sample for the current study included 21 young adults who lived with a sibling with depression while they were in high school. Participants ranged in age from 18 to 29 years (M = 22.39, SD = 3.47). The sample included nine men (42.86%) and twelve women (57.14%). Most of the participants were single (90.47%); only two were married and none had children. The criteria for participation were: (1) during adolescence, lived with a sibling with depression; (2) age 18–29 years old; (3) lived at home during adolescence (not at boarding school); (4) biological siblings with the same two parents (i.e., not foster care, to create a homogeneous group and reduce intervening variables). Exclusion criteria were: (1) participant with history of psychosis or schizophrenia; (2) participants currently expressing suicidal intent or depression.

### Data collection

Before the research began, the Ethics Committee of Oranim College approved the study, which was conducted in accordance with the Declaration of Helsinki (Authorization No. 117/2022). To maintain confidentiality, participants were referred to by assigned pseudonyms. The study was conducted via purposeful sampling, which aims to create a diverse sample reflecting different participants, thus providing a comprehensive picture of the topic being investigated [36]. We aimed to obtain a variety of background characteristics in terms of participants’ gender and ages within the range of participation criteria. We also sought an extensive geographic distribution to represent all localities in Israel. The interviewees were recruited via a letter posted on social media (e.g., Facebook). Participants responded by sending an email with their details. Interviewees were given a comprehensive explanation about the general research aims and after acknowledging having read the consent form, they gave their verbal informed consent. Interviews were conducted via Zoom from May to September 2022. The interviewers were female. One is a psychotherapist (PhD) and one an educational counsellor (MA). Both are experienced in conducting qualitative research. Participation was voluntary, and the interviewers promised to maintain participants’ anonymity and confidentiality. The interviews lasted 45–60 miniates.

The qualitative data in this study were collected via in-depth semi-structured interviews [36, 37]. The interview served as an instrument to help reveal and examine participants’ views and experiences. The interviews were conducted based on an interview guide that included significant key areas but was sufficiently flexible to allow for a dialogue to develop between interviewer and interviewee and to facilitate meaningful self-expression. The interview guide developed for this study was based on the literature review and on the clinical experience of two professionals who have worked with families coping with children with depression (Appendix 1). Each interview was recorded and then transcribed. Data collection proceeded until theoretical saturation was reached (i.e., additional interviews yielded no new material for analysis) [23].

### Data analysis

After data collection was completed, the researchers began analyzing the data. Data analysis encompassed the following stages: First, to achieve immersion and obtain a comprehensive understanding of the data, all information was read multiple times. Second, open coding was employed in that the researcher (I.L.) scrutinized each interview transcript line-by-line, while taking notes to identify the initial units of meaning emerging from the data and to facilitate naming subthemes. Third, the larger themes were reviewed by another researcher (M.L.), who then discussed them with I.L. Fourth, axial coding was applied, with the researchers gradually detecting context and content-related associations between themes and sub-themes. The researchers compared all the interviews to consolidate meanings and assigned names to the themes. They subsequently examined the interrelationships among the initial codes, sorting them into higher-order theoretical codes. Finally, the researchers integrated the results by conceptually reordering the core themes that arose from the data and placing them back into context, thus allowing for analysis and integration of large amounts of data and generation of abstractions and interpretations [36–38].

### Trustworthiness

Trustworthiness [39] was achieved in several ways. First, the interview material was transcribed verbatim, enabling the researchers to return to the original narrations. Each researcher then reviewed the material separately. The researchers engaged in ongoing reflection and continually bracketed their experiences and biases throughout all the study and analysis stages. The selected quotes represent most of the interview content and were translated from Hebrew to English. Each translation was verified by two native speakers, one of whom is a professional translator.

## Results

The qualitative analysis of the interviews yielded three main themes (Table 1):

**Table 1:** Classification of main theme and subcategories

Main theme	Subcategories
“School as a place of refuge”: The perspective of participants who grew up with a sibling with depression regarding their high school experience	• School was a place outside the home where the adolescent participants could rest from having to cope with their sibling’s illness. • Research participants reported behaving according to school norms in order to appease the environment and avoid conflict. • Research participants expected the school to be aware of their family situation and to help and support them, but their expectations were not met.
“I wanted the adults at school to see me”: Relations between research participants and the school educational staff	• Participants felt that the educational staff was unaware of and did not understand their family situation. • Many felt that the staff was distant, evasive, and arrogant. • A few reported that their relationship with the educational staff served as a source of support in coping with their sibling’s illness
“I was afraid people would relate to me as the sibling of a crazy person”: Participants’ relationships with their peers	• Participants concealed their sibling’s illness from their peers out of fear of negative stigmas, lack of understanding and a desire to maintain their sibling’s confidentiality. • Embarrassment and fear of social ostracism due to the sibling’s behavior. • Sense of relief and release after telling peers about sibling’s illness.

### Theme 1: “School as a place of refuge”: The perspective of participants who grew up with a sibling with depression regarding their high school experience

At first, most of the research participants described school as a “place of refuge”. On the surface, school served as a place where they could be themselves, have fun with friends, and temporarily leave the stormy situation at home behind. Some described the time they spent at school as a time when they turned into different people who were calmer and more relaxed.

School was my refuge. I really loved studying. I didn’t need much help from the school. Merely the fact that school was there helped me. I could distance myself, be with people who had nothing to do with what was going on at home. I could study there. There was even a music room with drums and keyboards and a guitar. The cocoon of school offered me a moment to run away from that entire story and be somewhere that was to some extent innocent and warm. (20-year-old male participant)

The research participants reported that they thought it was important to generate an image of being “perfect students” without any problems. They aspired for high academic achievements and tried to avoid behavior problems or friction with the educational staff. Some reported that during elementary school, before their sibling’s depression emerged, they had various problems, including behavioral problems, difficulty attending school regularly or social problems. Yet upon reaching high school they stopped asking their parents or teachers for help in coping with various issues related to school.

In elementary school my parents would insist and force me to get up and go to school, because I really hated going to school. But in high school and since my brother began showing signs of depression, I went to school without causing any problems. I also don’t know why it was so important for me to be in the top academic tracks and to get the highest grades. No one ever said anything to me or pressured me to do this, but I wanted to excel. (24 year old female)

Yet a closer look at the participants’ remarks paints a more complex picture. At first the participants described school as a “place of refuge” where they could escape the situation at home. But subsequently they described school as a place where no one noticed them, no one was aware of their distress, and no one answered their call for help. Phrases such as “no one cares” or “I didn’t tell anyone because no one would understand” were repeated in many of the interviews.

I never told anyone I needed help. I just didn’t see it that way. Everything seemed fine, my grades were good and I had friends and things were going well for me, so why should I ask for help? I also knew that if I admitted that things were not good for me I would begin feeling sorry for myself, and that was a red line for me. But in retrospect I know that if I had received some support things could have been a lot better for me and I would not have been ashamed of my sister. (24-year-old male)

### Theme 2: “I wanted the adults at school to see me”: Relations between research participants and the school educational staff

The research participants reported that they had limited contact with the school educational staff. They avoided revealing anything, engaging in emotional discussions, or developing meaningful relationships with them. Most stated that no one at school was aware of the situation at home caused by their sibling’s distress. Some indicated that at first they tried to tell an adult, but they felt the person did not understand them, forcing them to explain themselves a second time. This lack of understanding caused them to be suspicious of the professionals and to question their ability to help them.

I must not trust anyone. My job is merely to listen, to be there. I cannot trust anyone because everyone can break. Even those around me who appear to be the strongest break down, so if I depend on them or tell them something I will also break down. (22-year-old female participant)

Eliyahu, a 19-year-old male participant, reported that he avoided telling the educational staff about his family situation because he felt cut off from them, felt they were patronizing him and were powerless to help him.

Who can I tell? My teacher? And what exactly can she do? Give me a passing grade to appease me? Who can I talk to about this? With the school administration, which operates like the directorate of an industrial company? With the coordinator of my cohort, who tells you what to do even though she doesn’t even know your name? (25-year-old male participant)

In cases in which both siblings went to the same school, the educational staff only helped the sibling with depression. The research participants reported that they received no attention or special treatment. Except for a few cases, the school staff did not ask the research participants about their own situation or try to determine how their sibling’s illness affected them. Noa, a 29-year-old female participant, attended the same high school as her sister. She described feeling transparent and without any personal connection to the educational staff. Her teachers were aware of her sister’s illness, but often confused the two of them, so that her relationship with the teachers was impersonal, insensitive, and insulting.

The school was aware of my complex family situation. From time to time the counselor would call me aside to discuss what was going on at home. I always felt that she did not care. She would fill out a questionnaire and ask me a few questions but she did not really respond to what I said, except for saying that it sounded difficult. That’s where it ended, more or less. All she did was fill out the questionnaire she was supposed to complete and send me back to class. (20-year-old male participant)

Only two of the research participants remembered the educational staff as people who initiated discussions with them that improved their situation at home. They described educational counselors who were empathetic, patient and kind.

When I had homework that was really difficult and I was upset or worried about my brother and unable to concentrate, I knew I could go to the counselor’s office. Even just sitting there in her office would calm me down. She always welcomed me and let me know with her eyes that this is a place I can rest and cry. (21-year-old female participant)

### Theme 3: “I was afraid people would relate to me as the sibling of a crazy person”: Participants’ relationships with their peers

Many of the research participants noted that their friends were not aware of their sibling’s condition or of their stormy situation at home. They were sure they were the only ones coping with a difficult home situation, which made them socially deviant. Many were convinced that if they told their friends, who were unfamiliar with depression, they would not be able to cope. Hence, they expected their friends would not understand them, would pity them and would withdraw from them.

I didn’t tell any of my friends. Even today I don’t think that children would know how to cope with this. I certainly did not want anyone to feel sorry for me and I was afraid that if I told there would be some response, because it is hard for children to understand something like that. I didn’t want my classmates to talk about this. (23-year-old male participant)

In addition, the participants felt that if they told their friends, their friends would tell others and hence violate their sibling’s privacy. Therefore, they avoided revealing what was happening at home.

I had a really close friend. We were a twosome, and I really wanted to tell her but I knew I couldn’t…. I remember that her mother was very involved in the community and that if I told her she would tell her mother, who would tell everyone else…. Today I understand that not talking about this, not communicating about this, this fear—all this had an impact on me and on the support I could have received. (22-year-old female participant)

David, a 25-year-old male participant, articulately described the dilemma he faced because he attended the same school as his sister. On the one hand, he loved her and was concerned about her welfare. On the other hand, he was also concerned about his own social status and was afraid that his relationship with her would cause him to be rejected by his friends and labeled with a negative stigma.

Every situation that arose that was connected to my sister was potentially embarrassing. You hoped that no one would talk about it or connect her to you. In high school she was always looking for attention. That often placed me in uncomfortable situations. I must admit that when I entered the 11th grade and my sister left the school after completing 12th grade, I was relieved. (25-year-old male participant)

Four of the research participants reported that they relied on their friends for emotional support. These participants noted how much their friends’ support helped them make it through that period and feel secure. In retrospect, the research participants admitted that if other students had known what they were going through they could have helped them normalize their situation and alleviate their deep sense of loneliness.

I never spoke about what was going on at home until our annual field trip. One evening we sat with our counselor and lit candles. Each of us was supposed to choose something to illuminate. I said I wanted to illuminate my brother and the good things in life, and then I started crying. I ran away and couldn’t stop crying. My girlfriends sat with me that night and I told them everything. For four hours I talked and cried. I felt that the dam had burst. What a relief! (19-year-old female participant)

## Discussion

The current study sought to examine the high school experiences of young adults who lived with a sibling with depression during their adolescence. The participants described their complex relations with the school. Many claimed that school provided them an opportunity to escape the chaotic reality at home. Studies support the notion that siblings of individuals with mental health issues feel a heightened sense of responsibility and vigilance during a critical developmental stage [9, 16, 17, 20]. The present study posits that the participants may seek refuge within the school environment as a protective measure against potential harm, alienation, shame, thus restoring a sense of normalcy [25]. This concealment strategy may serve as a boundary reinforcement mechanism, consolidating and preserving the integrity of the family unit against external influences. Studies among siblings of people with mental disorders described how much they need independent activities that are not related to their involvement at home and do not include their siblings [40, 41].

At the same time as feeling that school is a place to hide, they also reported fantasizing about school serving as a place where they could receive comfort. According to Winnicott [42], “It is a joy to be hidden, and disaster not to be found.” The research participants used school as a place of refuge where they could overlook their troubles at home and not have to cope with them. Yet their wish to be found was not realized. This finding is in line with research conducted among siblings and parents of children with mental disorders and chronic illnesses [43]. Studies have shown that although many teachers are willing to assume responsibility for caring for students’ mental health, they may lack knowledge about symptoms and the strategies to manage them effectively [44].

In the current study the participants behaved in a quiet and placating manner, and they avoided conflicts at school. According to Bowen [31], the participants adopted behavior characterized by fusion in order to survive their family situation. That is, they internalized the needs of others to the point of self-nullification. Habits the participants adopted at home, such as avoiding telling others about their feelings and denying personal needs, also characterized their behavior at school. As a result of similar patterns at school and at home, the research participants were not the center of attention in either place.

In addition, they may have felt a sense of responsibility and a need to help their family rather than adding to their problems by causing academic issues [45]. One explanation is that because they were good and quiet students and did not cause problems at school, the school staff was concerned with other things [46]. Another explanation is that school staff members who must deal with such issues are confronted with the circumstances of their own personal world. Encountering similar stories can arouse memories, cause emotional flooding and lead to a sense of helplessness, thus engendering distancing and avoidance behavior [30].

In the current study, the participants described their fears that their friends would find out about their sibling with depression. This finding is in line with the findings of previous studies showing that the family members of people with mental illnesses try to avoid divulging their family situation due to shame, fear of being stigmatized and fear of negative consequences [14, 28]. The literature contains a great deal of evidence showing that people coping with mental illness are not the only ones who face society’s negative stigmas. Family members also must cope with negative labeling, or what the literature refers to as “stigma by association” [14, 47], according to which family members bear responsibility for their relative’s illness and are even considered to be at blame. Family members who internalize and believe this stigma report a high degree of psychological distress, low self-esteem and low self-confidence [14, 47].

A healthy portion of shame helps individuals protect themselves through deterrence and creates a correct balance of self-exposure [14]. But when the feeling of shame intensifies, becomes complicated, and takes over at the expense of other feelings, it has a negative and inhibiting effect [49]. Among families of victims, shame “shatters” the set of family expectations and sometimes even the image of an “ideal” family [48, 49]. Many times, such individuals feel that “a stranger cannot understand this.“ Fear of reactions that are judgmental, intrusive, and critical reactions as well as of those that are excessively curious to the point of “voyeurism” makes it difficult to trust anyone and reveal the “secret” [48, 49]. In extreme cases, the family unit may become socially isolated.

In this study, the few participants who were able to share their story with someone else felt significant relief. Growing up with a sibling who has depression can have a profound impact on an adolescent’s school experiences. On the one hand, having a sibling with depression can raise awareness of and elicit empathy for mental health issues and encourage the adolescent to prioritize self-care and seek support when needed. On the other hand, living with a sibling with depression can also introduce stress and anxiety into the adolescent’s life, lead to distractions, and hinder academic performance. Moreover, these adolescents may feel pressured to provide their sibling ongoing support and keep the family dynamic functioning, which can take a toll on their own mental health and success at school. The burden of care for children with chronic diseases generally falls upon parents and siblings, who must balance the affected child’s healthcare needs with other commitments. A meta-analysis of the siblings of children with chronic diseases showed that they are at risk for several negative effects [50]. They may find that their parents give them less time and attention. Such disruptions to family life may influence the psychosocial well-being of all family members and thus have an impact on the demand for health services.

On the other hand, growing up with a sibling with a mental illness has been shown to have numerous positive effects, such as developing tolerance, sensitivity, the ability to work independently, high self-confidence, and leadership abilities [6, 51]. This growth resulting from stressful situations is known as post-traumatic growth, such that individuals find meaning in difficult experiences and discover their personal abilities have improved compared to their situation before the crisis [52]. In fact, research participants facing traumatic life realities have managed to grow, distilling the advantages of the crisis that befell their family and discovering new strengths within themselves.

When depression emerges in a family, family members often experience it as an extreme and destabilizing event [14]. Mental illness shares the characteristics of both chronic and acute conditions, posing a threat to individual and family integrity and causing emotional distress. Nevertheless, this experience can also lead to growth and strengthening of the individuals and the entire family unit. During adolescence, teenagers want to feel “like everyone else” and use school and friends as a refuge from the difficulties at home [21]. Professionals must approach these individuals with great sensitivity and recognize their different needs.

### Implications

The results of the current study have implications for school psychologists, counselors, and social workers. These professionals need to understand that growing up with a sibling who has depression can potentially affect adolescents’ school experiences. Hence, they should strive to create a safe and inclusive classroom environment, encourage open communication, and provide a non-judgmental space for students to express their feelings and experiences. They should be mindful of the emotional well-being of these students, check up on them regularly, and provide them with resources and support if they are struggling. They should strive to foster a sense of community and connectedness, encourage students to form supportive relationships with their peers, and provide opportunities for them to connect with others who may have similar experiences. Every student is unique, and each one should be treated with empathy and understanding. By providing support and resources, professionals can help students navigate the challenges that come with growing up with a sibling who has depression and thus foster their academic and personal growth. In addition, it is important for professionals to maintain contact with parents and the entire family as a system and to pay particular attention to adolescents living with a sibling with depression.

### Limitations

To the best of our knowledge, no other published studies have explored the experiences and insights of young adults who lived with a sibling with depression during their adolescence. The study’s methodological strength lies in its construction of a homogeneous group that includes clear inclusion criteria, specifically the siblings of those afflicted with a single disorder, in contrast to studies that included a variety of mental disorders.

Nevertheless, the study has several limitations. Due to the nature of qualitative research, the sample size of this study was limited. Moreover, this study focused on a specific group of participants who as adolescents had a sibling affected with depression. Therefore, the results cannot be generalized to their experiences later in life. Another limitation is that the study used a convenience sample of individuals who self-identified themselves as having a sibling with depression.

The study was conducted during a period when the COVID-19 pandemic in Israel was on the decline. Nevertheless, the pandemic may have affected the emotional state of the participants as well as of the educational teams they described. In addition, the study focused on the participants themselves. In order to obtain a more comprehensive picture we recommend also examining the perspectives of the parents, of the siblings affected by depression, and of the educational teams. In addition, to preserve confidentiality, we refrained from inquiring about the names of the schools or the identities of the staff members. It is possible that some of the participants attended the same school as their affected sibling, though we did not examine this. Nevertheless, we included a broad nationwide sample of participants. Finally, many factors were unexplored in this study, among them the effects of age, gender, family structure, that would be of interest to investigate in future research.

## Conclusion

This study examined the experiences of young adults who retrospectively reported on their high school experiences growing up with a sibling with depression. The research participants reported feeling isolated during high school and stated that those around them did not understand them. Many participants indicated that they sought to appease their surroundings through positive behavior that conformed to the school’s behavioral norms. In addition, they made efforts to conceal their family situation, thus making it impossible for them to receive support from their peers and from the school educational staff, despite needing help and support during that period.

## Electronic supplementary material

Below is the link to the electronic supplementary material.

Supplementary Material 1

## Data Availability

The datasets generated and analyzed during the current study are not publicly available due to privacy concerns but are available from the corresponding author on reasonable request.
