# Supplementary material for: “I wanted to hide but also to be found”: the high school experiences of young adults who grew up in the same home as a sibling with depression
Source: BMC Psychol. 2023 Jun 29;11:190. doi: 10.1186/s40359-023-01234-y (PMC10311711; doi:10.1186/s40359-023-01234-y)
Supplement: Supplementary file 1 — Supplementary Material 1 [file 40359_2023_1234_MOESM1_ESM.docx]

*Appendix 1: Interview questions posed to young adults who grew up in the same home as a sibling with depression.*

| 1 | How do you define depression? |
| --- | --- |
| 2 | Tell me about your sibling’s experience with depression. |
| 3 | What difficulties did you face growing up with a sibling who has depression? |
| 4 | From your point of view, did your performance at school change after your sibling was diagnosed? |
| 5 | Have you experienced emotional or social changes? Can you provide an example? |
| 6 | Did the educational staff know about the situation at home? |
| 7 | What support did you get and what support did you need? |
| 8 | Did you share your home situation with your friends? What were their reactions? How did that make you feel? If you didn't share, can you discuss the concerns you had? |
| 9 | What advice would you give to educators working with students who have siblings with depression? |
| 10 | Is there anything else you would like to tell me, which was not covered in the questions above? |
